# B7-H3 promoted proliferation of mouse spermatogonial stem cells *via* the PI3K signaling pathway

**DOI:** 10.18632/oncotarget.23457

**Published:** 2017-12-20

**Authors:** Xuedong Wei, Kai Li, Guangbo Zhang, Yuhua Huang, Jinxing Lv, Miao Li, Lun Zhao, Caibin Fan, Jinxian Pu, Jianquan Hou, Hexing Yuan

**Affiliations:** ^1^ Department of Urology, First Affiliated Hospital of Soochow University, Suzhou, Jiangsu, People’s Republic of China; ^2^ Department of Urology, Suzhou Municipal Hospital, Suzhou, Jiangsu, People’s Republic of China; ^3^ Department of Clinical Immunology Laboratory, First Affiliated Hospital of Soochow University, Suzhou, Jiangsu, People’s Republic of China

**Keywords:** B7-H3, testis, mouse spermatogonial stem cell, proliferation, PI3K pathway, Immunology

## Abstract

**Objective:**

We found seminal B7-H3 was associated with human sperm concentration. However, the mechanism is unclear. The purpose of this study was to investigate the expression of B7-H3 in mouse testis and determine the effects of B7-H3 on the proliferation of mouse spermatogonial stem cells (SSCs) and the underlying mechanisms.

**Methods:**

B7-H3 expression in the testis of mice at different ages (3 weeks, 8 weeks, 4 months and 9 months) was detected by western blot and immunohistochemistry. CCK-8 were used to measure mouse SSCs proliferation after incubation with different concentrations of B7-H3 for 1-72 h *in vitro*. Flow cytometry was used to analyze the cell cycle of mouse SSCs after incubation with different concentrations of B7-H3 for 48 and 72 h. The signaling pathways involved were assessed by western blot.

**Results:**

Four-month-old mice had the highest expression of B7-H3 in the testis, while 3-week-old mice had the lowest expression of B7-H3. B7-H3 was predominantly detected on the membrane and in the cytoplasm of Sertoli cells. Furthermore, B7-H3 promoted mouse SSCs proliferation and increased the percentage of cells in S+G2/M phase in a time- and dose-dependent manner *in vitro*. These effects were inhibited by LY294002, indicating the involvement of the phosphoinositide 3-kinase signaling pathway.

**Conclusions:**

The expression of B7-H3 in mouse testis, especially Sertoli cells, was associated with mouse age. *In vitro*, B7-H3 promoted the proliferation and accelerated the cell cycle of mouse SSCs via the PI3K pathway, indicating a critical role of B7-H3 expressed by Sertoli cells in mouse spermatogenesis.

## INTRODUCTION

Spermatogenesis is a continuous, productive and strictly controlled process [1, 2] that is supported by the self-renewal and differentiation of spermatogonial stem cells (SSCs) in the microenvironment of the seminiferous tubules [3]. Sertoli cells, the only somatic cell type in the tubules, control the proliferation and differentiation of SSCs by direct interaction and secretion of specific factors [4].

B7-H3, a type I transmembrane protein, was initially described as a new member of the B7 family in 2001 [5]. There is a soluble form of this protein, which can be released into the circulation [6]. We previously reported that soluble B7-H3 was detected in expressed prostatic secretions [7]. To date, the B7-H3 protein has been detected in multiple organs, including the liver, testis, and epididymis [8]. However, there is no consensus on the biological functions of B7-H3. The original study reported that B7- H3 could activate T cells and increase T cell proliferation as well as interferon-γ (IFN-γ) production [5]. In contrast, several subsequent studies found that B7-H3 inhibited T cell function [9–12]. Other reports have examined the non-immunological role of B7-H3. Sun et al. [13] found that B7-H3 inhibited tumor growth by decreasing the expression of vascular endothelial growth factor (VEGF). B7-H3 also promoted the differentiation of human marrow stromal cells to osteoblasts [14]. Our previous study identified an association between seminal B7-H3 with sperm concentrations and progressive motility and also showed that B7-H3 promoted human sperm motility *in vitro* [15]. Thus, B7-H3 may have a critical role in normal spermatogenesis. However, the underlying mechanisms and the biological functions of B7-H3 in this process are still unknown.

Extracellular signal-Regulated Kinases (ERK), c-Jun N-terminal kinase (JNK) and Phosphoinositide 3-Kinases (PI3Ks) are the three most intensively studied signaling pathways that participate in spermatogenesis [16–19]. PI3Ks are ubiquitous lipid kinases that serve as signal molecules downstream of receptors on the cell surface [20, 21]. To date, the PI3K signaling pathway has been shown to play a role in male fertility and spermatogonia proliferation. Feng et al. [22] reported that stem cell factor (SCF)/C-kit promoted proliferation of type A spermatogonia by recruiting PI3K/AKT/p70 S6 kinase (p70S6K)/cyclin D3. Sagare-Patil et al. [23] found that progesterone regulated motility and hyperactivation of human sperm *via* the PI3K-AKT pathway. Moreover, glial cell line-derived neurotrophic factor (GDNF) utilizes the rat sarcoma (Ras)/ERK1/2 pathway to promote SSC proliferation [24]. MAPK/ERK kinase (MEK)/ERK signaling contributes to periodic self-renewal/proliferation of SSCs [25]. Hydrogen peroxide induced reactive oxygen species (ROS) *via* activation of the p38 mitogen-activated protein kinase (MAPK) and JNK pathways to enhance self-renewal of SSCs [26]. The JNK1/2 pathway may play an important role in regulating sperm viability [27].

Based on these reports and our previous studies mentioned above, we examined (1) whether B7-H3 expression in mouse testis is dependent on age or gonad maturation; (2) whether B7-H3 regulates the proliferation and cell cycle of mouse SSCs; and (3) which signaling pathways are involved in this process.

## RESULTS

### Localization of B7-H3 in mouse testis

**Figure 1:**
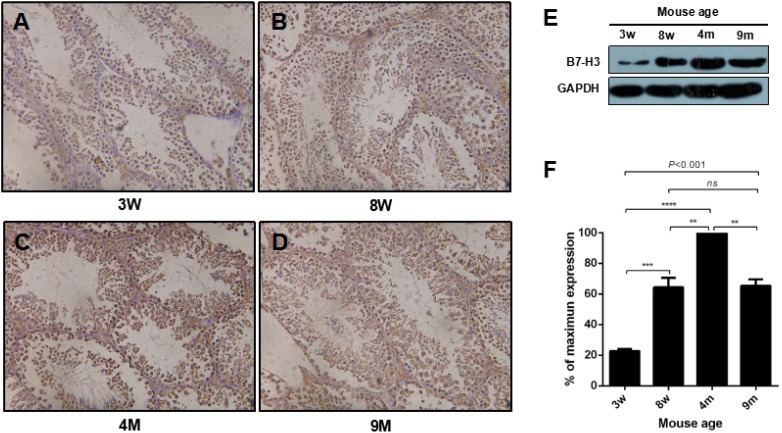
The expression of B7-H3 in mouse testis at different ages **A.** to **D.** Representative images of mouse testis tissues stained with B7-H3. Magnification, X200. **E.** The collected mouse testes from different ages were digested and lysed, and the precleared tissue lysates were assessed by western blot using an anti-B7-H3 antibody. GAPDH was used as the loading control. **F.** The graph represents the quantification of B7-H3 abundance shown in (*E*) normalized to the corresponding GAPDH in three independent experiments. The results are expressed as the mean±standard deviation (*n* = 3). Differences were analyzed by one-way ANOVA followed by Tukey's post hoc analysis. **P < 0.01, ***P < 0.001 and ****P < 0.0001.

To examine B7-H3 in the different phases in mouse sexual maturity, as described by Flurkey et al. [28], we selected four groups of mice: 3-w-old mice, 8-w-old mice, 4 m-old mice and 9-m-old mice, which represented juvenile, adolescent, mature and middle aged mice. As shown in Figure 1A to 1D, compared to the sexually immature mice, the sexually mature mice had increased amounts of sperm in the seminiferous tubules. B7-H3 staining was positive both on the membrane and in the cytoplasm of mouse testis tissues from all stages. The testes from the 4 m mice had the strongest B7-H3 staining compared with the staining of those from other ages.

### The expression of B7-H3 in mouse testes at different stages

The highest expression of B7-H3 was observed in the testes of the 4 m mice (all *P* < 0.01), which were sexually mature. The expression levels of B7-H3 in the mouse testes of the 3 w, 8 w and 9 m groups were 22.76%±1.31%, 64.40%±6.25% and 65.36%±4.20%, respectively, compared to those of the 4 m mice (Figure 1E and 1F). These data also demonstrated that the expression of B7-H3 in testis was increased during the process of maturation to an adult, after which it decreased.

### B7-H3 promotes mouse SSC proliferation and cell cycle progression

**Figure 2:**
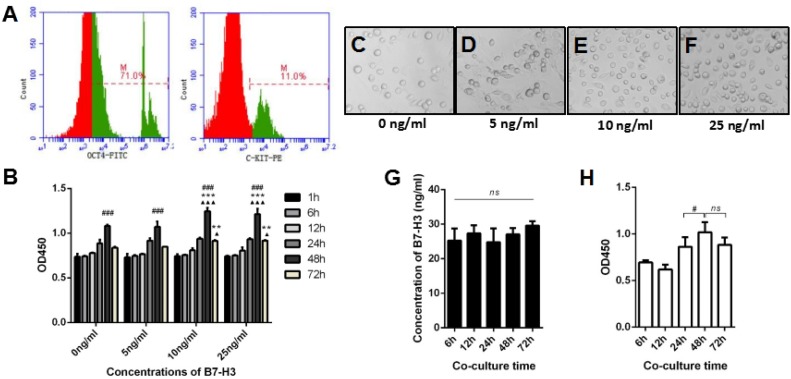
The effect of B7-H3 on mouse SSC proliferation **A.** The expression of C-kit and Oct-4 on mouse SSCs was analyzed by flow cytometry. **B.** Mouse SSCs were incubated with different concentrations of B7-H3 (0, 5, 10, 25 ng/mL) for various time points (1, 6, 12, 24, 48, 72 h) *in vitro*. Then, cells were collected for proliferation assessment using CCK8 assays. The results are expressed as the mean±standard deviation (*n* = 3). Differences were analyzed by two-way ANOVA followed by Tukey's post hoc analysis. ###P < 0.001 *versus* 24 h. **P < 0.01, ***P < 0.001 *versus* 0 ng/ml. ▲*P* < 0.05, ▲▲▲*P* < 0.0001 *versus* 5 ng/ml. **C.** to **F.** Representative images of SSCs incubated with different concentrations of B7-H3 at 48 h. **G.** The SSCs were co-cultured with TM4 for 6 to 72 h *in vitro*, and the supernatants were collected at each time point for B7-H3 evaluation. The data were analyzed by repeated measures one-way ANOVA. **H.** The co-cultured SSCs in (*G*) were harvested and assessed for proliferation as described in (*B*). Data were analyzed by repeated measures one-way ANOVA with Tukey's post tests. #*P* < 0.05 *versus* 24 h.

Mouse SSCs were used to investigate the effects of B7-H3 on spermatogenesis. Figure 2A shows representative flow cytometry data of C-kit and Oct-4, markers for SSCs [29–31], and expression on mouse SSCs (Figure 2A).

To investigate the role of B7-H3 in mouse SSC growth, we analyzed the proliferation rate of the cells using CCK-8 assays *in vitro*. Mouse SSCs were incubated with different concentrations of B7-H3 (0, 5, 10, 25 ng/mL) for various time points (1, 6, 12, 24, 48, 72 h). As shown in Figure 2B, regardless of the concentration of B7-H3, from 1 to 48 h, the proliferation rate of SSCs increased; however, from 48 to 72 h, the proliferation rate of SSCs decreased. After 48 h of incubation, the proliferation rates of SSCs treated with 10 ng/ml (OD value, 1.25±0.04) and 25 ng/ml (OD value, 1.21 ±0.06) B7-H3 were both significantly higher than those of SSCs treated with 0 ng/ml (OD value, 1.08±0.02) and 5 ng/ml (OD value, 1.07±0.06) B7-H3 (all *P* < 0.001). However, the differences in cell proliferation between the 0 ng/ml and 5 ng/ml B7-H3-treated groups were not statistically significant (*P* > 0.05). Moreover, there was no difference in cell proliferation between SSCs treated with 10 ng/ml and 25 ng/ml B7-H3 (*P* > 0.05).

Similarly, after 72 h of incubation, cell proliferation in the 10 ng/ml (OD value, 0.91±0.01) and 25 ng/ml (OD value, 0.92±0.01) B7-H3-treated groups was higher than that in the groups treated with 0 ng/ml (OD value, 0.84±0.01) and 5 ng/ml (OD value, 0.85±0.00) B7-H3 (all *P* < 0.05). Furthermore, the differences between the 10 ng/ml and 25 ng/ml B7-H3-treated groups were not statistically significant (*P* > 0.05). These results indicated that B7-H3 promotes SSC proliferation in a time- and dose-dependent manner.

**Figure 3:**
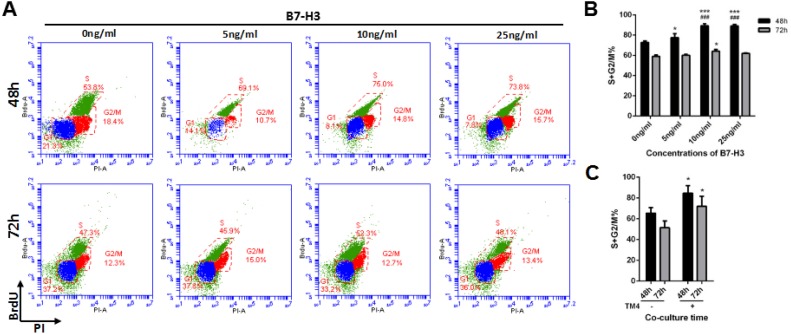
The effect of B7-H3 on the cell cycle of mouse SSCs **A.** Mouse SSCs were incubated with different concentrations of B7-H3 (0, 5, 10, 25 ng/mL) for 48 and 72 h *in vitro*, and the cell cycle was assessed by BrdU incorporation. Representative flow cytometry data from three independent experiments are shown. **B.** The graph represents the percentage of cells in S+G2/M shown in (*A*) in three independent experiments. The results are expressed as the mean±standard deviation (*n* = 3). Differences were analyzed by two-way ANOVA followed by Tukey's post hoc analysis. *P < 0.05, ***P < 0.001 *versus* 0 ng/ml. ###*P* < 0.001 *versus* 5 ng/ml. **C.** The SSCs were co-cultured with or without TM4 for 48 or 72 h and collected for cell cycle analysis. Data were analyzed by two-way ANOVA, followed by Tukey's post hoc analysis. *P < 0.05 *versus* non-co-cultured cells.

Additionally, the cell cycle was evaluated by using BrdU incorporation after mouse SSCs were incubated with different concentrations of B7-H3 for 48 and 72 h. As shown in Figure 3B, after 48 h of incubation, the percentage of SSCs in S+G2/M phase was significantly higher in the 10 ng/ml (89.20%±2.07%) and 25 ng/ml (89.00%±1.61%) B7-H3-treated groups than that in the groups treated with 0 ng/ml (72.77%±1.53%) and 5 ng/ml (77.43%±4.01%) B7-H3 (all *P* < 0.001). However, the differences between the 10 ng/ml and 25 ng/ml B7-H3-treated SSCs were not statistically significant (*P* > 0.05). After 72 h of incubation, only the 10 ng/ml B7-H3-treated SSCs had a higher S+G2/M percentage compared to that of cells treated with 0 ng/ml B7-H3 (*P* < 0.05).

Since B7-H3 was predominantly expressed on mouse Sertoli cells but not on SSCs (Supplementary Figure 1), we co-cultured these two cell types and investigated if the Sertoli cell-derived B7-H3 affects the proliferation of SSCs. As shown in Figure 2G, the concentrations of B7-H3 in co-culture supernatants at 6, 12, 24, 48, and 72 h were 25.30±3.45, 27.35±2.30, 24.82±3.96, 27.07±1.82 and 29.62±1.27 ng/ml, respectively, which were higher than those used in the current study. However, no significant difference was found between these time points (*P* > 0.05). The SSCs co-cultured with TM4 for 48 h had a higher proliferation rate than those co-cultured for 24 h (*P* < 0.05), consistent with the data from B7-H3 protein stimulation. However, there was no difference between the 48 and 72 h co-cultures (*P* > 0.05) (Figure 2H). Co-culture with TM4 promoted the cell cycle of SSCs at both 48 h and 72 h (*P* < 0.05), although the difference between these two time points was not significant (*P* > 0.05) (Figure 3C).

### B7-H3 activates the PI3K pathway in mouse SSCs

**Figure 4:**
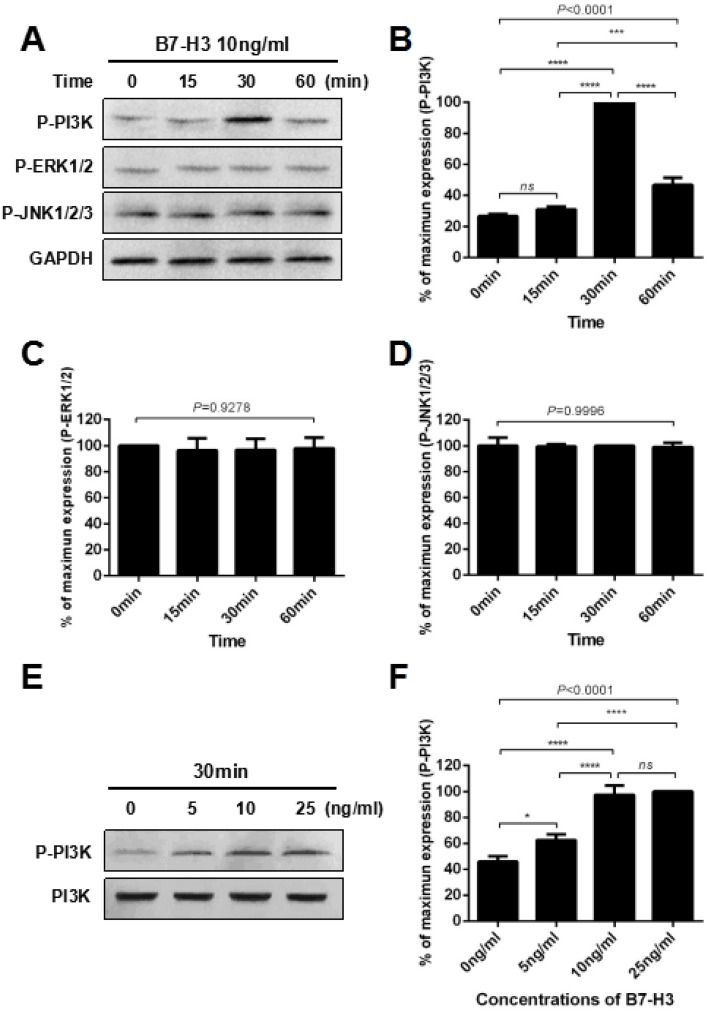
B7-H3 activated the PI3K signaling pathway in mouse SSCs **A.** Mouse SSCs were incubated with 10 ng/ml B7-H3 for 0, 15, 30 and 60 min *in vitro*, and cell lysates were assessed by western blot using anti-phospho-PI3K, anti-phospho-ERK1/2 and anti-phospho-JNK1/2/3 antibodies. GAPDH was used as a loading control. **B.** to **D.** Graphs represent the quantification of phosphorylation of PI3K, ERK1/2 and JNK1/2/3 shown in (*A*), respectively, which was normalized to the corresponding GAPDH in three independent experiments. **E.** Mouse SSCs were incubated with different concentrations of B7-H3 (0, 5, 10, 25 ng/ml) for 30 min *in vitro*, and cell lysates were assessed by western blotting using an anti-phospho-PI3K antibody. PI3K was used as the loading control. **F.** The graph represents the quantification of phosphorylation of PI3K shown in (*E*), which was normalized to the corresponding PI3K in three independent experiments. The results are expressed as the mean±standard deviation (*n* = 3). Differences were analyzed by one-way ANOVA followed by Tukey's post hoc analysis. *P < 0.05, ***P < 0.001, ****P < 0.0001.

As shown in Figure 4A, mouse SSCs were treated with 10 ng/ml B7-H3 at various time points (0, 15, 30, 60 min), and the lysates were analyzed by western blotting. The results indicated that B7-H3 substantially increased the phosphorylation of PI3K (P-PI3K) at 30 min compared to that at other time points (all *P* < 0.0001, Figure 4A and 4B). ERK and JNK, the other two commonly studied signaling pathways involved in regulating SSC proliferation, were also evaluated. However, there were no significant changes in the phosphorylation of ERK1/2 (P-ERK1/2) (*P* = 0.9278) and JNK1/2/3 (P-JNK1/2/3) (*P* = 0.9996) after treatment with B7-H3 for different times.

To further explore the effect of B7-H3 on phosphorylation of PI3K in mouse SSCs, we incubated the cells with different concentrations of B7-H3 (0, 5, 10, 25 ng/ml) for 30 min. As shown in Figure 4E and 4F, the 25 ng/ml B7-H3-treated SSCs had the highest phosphorylation of PI3K. However, the difference was not statistically significant compared to that of the 10 ng/ml B7-H3-treated SSCs (*P* > 0.05). Nevertheless, both of these two groups had higher phosphorylation of PI3K than that of cells treated with 0 ng/ml and 5 ng/ml B7-H3 (all *P* < 0.0001).

### A PI3K inhibitor blocks the progression of the cell cycle of SSCs induced by B7-H3

To further confirm the role of the PI3K pathway in the B7-H3-induced cell cycle progression of mouse SSCs, we pretreated the SSCs with 10 μM LY294002, a widely used PI3K inhibitor, prior to incubation with 10 ng/ml B7-H3 for 48 h and 72 h.

**Figure 5:**
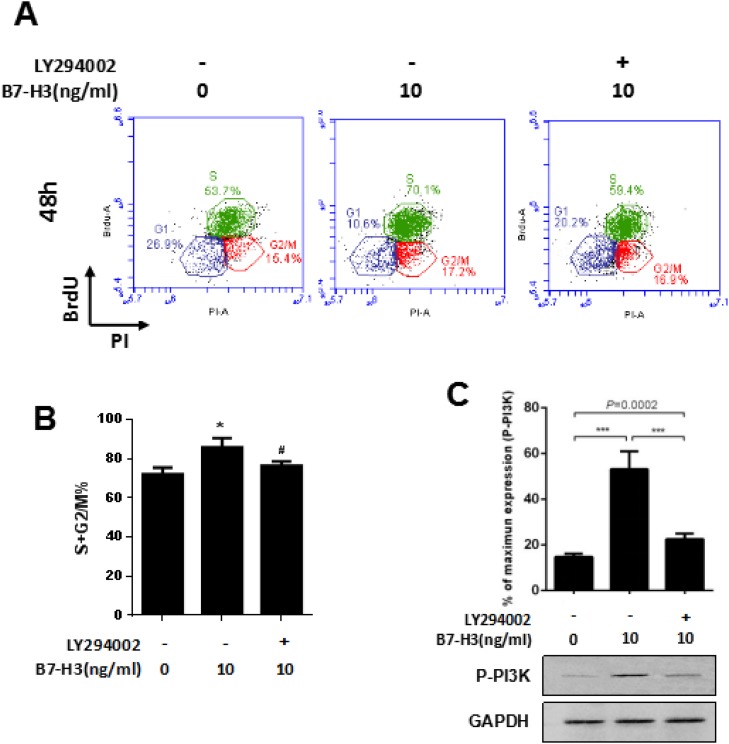
A PI3K inhibitor attenuated the B7-H3-induced cell cycle promotion of SSCs **A.** Mouse SSCs were pretreated with 10 μM LY294002 before incubation with 10 ng/ml B7-H3 for 48 h *in vitro*, and the cell cycle was assessed by BrdU incorporation. Representative flow cytometry data of three independent experiments are shown. **B.** The graph represents the percentage of S+G2/M shown in (*A*) in three independent experiments. The results are expressed as the mean±standard deviation (*n* = 3). Differences were analyzed by one-way ANOVA followed by Tukey's post hoc analysis. *P < 0.05 *versus* no LY294002 inhibition and B7-H3 stimulation. #*P* < 0.05 *versus* with B7-H3 stimulation but without LY294002 inhibition. **C.** Cells treated the same as in (*A*) were also assessed by western blotting for phosphorylation of PI3K. The lower membrane shows the immunoblotting of phosphorylated PI3K in SSCs. The upper graph shows the quantification of phosphorylated PI3K normalized to the corresponding GAPDH in three independent experiments. The results are expressed as the mean±standard deviation (*n* = 3). Differences were analyzed by one-way ANOVA followed by Tukey's post hoc analysis. ***P < 0.001.

After 48 h of incubation, the percentage of S+G2/M cells in the 10 ng/ml B7-H3-treated SSCs (85.77%±4.69%) was significantly increased compared to that in SSCs without B7-H3 treatment (72.13%±3.33%) (*P* < 0.05), while the S+G2/M percentage was significantly decreased in those SSCs treated with both LY294002 and B7-H3 (76.23%±2.20%) (*P* < 0.05) (Figure 5A and 5B). Meanwhile, western blot analysis showed that PI3K phosphorylation was successfully inhibited by LY294002, which indicated that PI3K was the pathway involved in the B7-H3-induced cell cycle progression.

## DISCUSSION

In mammals, sperm is produced in the seminiferous tubules of the testis [2], which are composed of seminiferous and interstitial tissues. Sertoli cells, the only sustentacular cells in the interstitial tissues, play a critical role in spermatogenesis by secreting specific substances and direct cell-cell interactions with SSCs. In 2004, Suh et al. [8] first detected B7-H3 expression in mouse testis. Recently, our studies showed that there was a high concentration of soluble B7-H3 in human expressed prostatic secretions [7] as well as in seminal plasma [15]. Additionally, the levels of seminal B7-H3 were associated with sperm counts. Thus, several interesting questions were raised: where B7-H3 localizes in the testis; whether the expression of B7-H3 is associated with sexual maturity; whether this protein influences SSC proliferation and what is the underlying molecular mechanism.

The relative rates of development, maturation and aging in mice and humans are very different. Few published reports have examined the age-specific milestones for mouse sexual maturity. However, Flurkey et al. [28] identified three stages of adult maturation and aging in mice, in which 3-6 months represents the mature adult stage without senescence. Then, the mice enter a middle aged stage from 10 to 15 months. In this stage, degeneration of organs and tissues is initiated. In the current study, we selected 3 w, 8 w, 4 m and 9 m mice, which represented juvenile, adolescent, mature and middle age stages, respectively. We detected B7-H3 expression in the testis of the 3 w, 8 w, 4 m and 9 m mice by IHC and western blot. Our data demonstrated that the B7-H3 protein was predominantly localized on the membrane and in the cytoplasm of Sertoli cells but was not expressed on the surface of mouse SSCs (Supplementary Figure 1). The western blot results showed alterations of B7-H3 expression with mouse age. The 4 m mice had the highest expression of B7-H3, while the 3 w mice had the lowest expression. A decrease in B7-H3 expression was observed in 9 m mice compared to that of mice in adulthood (4 m). These findings indicated that B7-H3 protein, which is expressed by Sertoli cells, peaked when mice were sexually mature and may play a role in spermatogenesis.

Because SSC proliferation is a critical step in spermatogenesis, we investigated the effect of soluble B7-H3 on the proliferation and cell cycle of mouse SSCs. We isolated SSCs from C57BL/6 mice and measured C-kit and Oct-4 expressions in these cells by flow cytometry. Our results showed that the isolated cells weakly expressed C-kit and strongly expressed Oct-4, which was consistent with previous reports about SSC surface marker expression [1, 29, 30, 32].

The CCK-8 data showed that B7-H3 promotes SSC proliferation in a time- and dose-dependent manner. We also found that B7-H3 promoted cell cycle progression by increasing the percentage of S+G2/M phase cells. These results indicated that B7-H3, similar to other substance secreted by or expressed on Sertoli cells, promotes mouse SSC proliferation, which is consistent with our previous findings that the levels of seminal B7-H3 were associated with sperm counts in humans. Although these data suggested that the B7-H3 protein positively affected the proliferation of mouse SSCs, and B7-H3 is predominantly expressed by Sertoli cells *in vivo*, we investigated whether SSCs were affected by Sertoli cell-derived B7-H3. Then, we co-cultured mouse SSCs with TM4 cells, a Sertoli cell line, in an *in vitro* transwell system. Our data demonstrated that in the co-culture system, TM4 expressed sufficient levels of B7-H3 to promote the proliferation and cell cycle of mouse SSCs, confirming the important role of Sertoli-derived B7-H3 in spermatogenesis.

To further clarify the possible molecular mechanism underlying B7-H3-enhanced SSC proliferation, we investigated three intensively studied signal pathways involved in SSC proliferation [22, 24, 26]. The phosphorylation of PI3K in mouse SSCs was significantly increased after 30 min of incubation with 10 ng/ml B7-H3 *in vitro*, while the phosphorylation of ERK and JNK was not increased. Moreover, the B7-H3-induced phosphorylation of PI3K reached a plateau, with a saturated concentration of 10 ng/ml. To confirm these results, we added LY294002, a widely used PI3K inhibitor [33, 34], to SSCs prior to incubation with B7-H3. As expected, the B7-H3-induced activation of PI3K and the increase in S+G2/M cell percentage of SSCs were both attenuated. These data suggested that B7-H3 promoted mouse SSC proliferation *via* the PI3K pathway.

This study demonstrated for the first time that B7-H3 promoted mouse SSC proliferation through the PI3K pathway, revealing the possible mechanism of B7-H3 in spermatogenesis and identifying B7-H3 as a potential therapeutic agent for human oligozoospermia treatment. However, several limitations of the current study should be noted. First, all the data were generated from mouse testis because it is difficult to obtain testis tissues from healthy humans. Second, although mice have been extensively used in scientific research for decades, few papers have reported the standard for age group determination compared to that of humans. Third, because the biological function of B7-H3 is unknown, only three signaling pathways were selected for analysis in the current study. Although the PI3K pathway was identified as a transducer of B7-H3 signaling in mouse SSCs, the current data are preliminary, and the underlying mechanism requires further analysis. Finally, to confirm the constructive role of Sertoli cell-derived B7-H3 in spermatogenesis, selective B7-H3 blocking or a knockdown model should to be studied.

## MATERIALS AND METHODS

### Animals and reagents

C57BL/6 mice were purchased from Xi Nuosai Biotechnology (Suzhou, CHN). Mouse embryonic fibroblasts (MEFs) were obtained from the Cell Bank of the Chinese Academy of Sciences (Shanghai, CHN). Mouse Sertoli cells (TM4) were purchased from Sai Baikang Biotechnology (Shanghai, CHN). Anti-PI3K antibody was obtained from Cell Signaling Technology (Boston, USA). Recombinant mouse B7-H3 was purchased from R&D Systems (Minneapolis, USA). Anti-phospho-PI3K antibody, anti-phospho-ERK1/2 antibody, anti-phospho-JNK1/2/3 antibody, anti-C-kit antibody, anti-Oct-4 antibody, FITC-conjugated anti-B7-H3 antibody, PE-conjugated goat anti-rat IgG, BRDU, anti-BrdU antibody and Alexa Fluor 488- conjugated goat anti-rat IgG were obtained from Abcam (Cambridge, USA). Anti-B7-H3 antibody and goat anti-rabbit IgG antibody were purchased from Lianke Biotechnology (Hangzhou, CHN). FITC-conjugated goat anti-rabbit IgG was purchased from Proteintech Group (Chicago, USA). Recombinant murine LIF and recombinant murine GDNF were purchased from Peprotech Inc. (Rocky Hill, USA). Fetal bovine serum (FBS) and B27 were purchased from Invitrogen (Carlsbad, USA). Phosphate-buffered saline (PBS), high-glucose DMEM and penicillin/streptomycin were obtained from HyClone (Logan, USA). Anti-GAPDH antibody, type IV collagenase, 0.25% trypsin, insulin, transferrin and Triton X-100 were purchased from Sigma (Saint Louis, USA). Mitomycin C was obtained from Roche (Basel, CH). Cell Counting Kit-8 (CCK-8) was purchased from Dojindo Molecular Technologies (Kumamoto, JP). The PI3 kinase inhibitor LY294002 was obtained from Selleck Chemicals (Houston, USA).

### Mice and sample preparation

The protocols for the mouse experiments were approved by the Animal Ethics Committee of Soochow University. For each independent western blot, we obtained the testes from 4 C57BL/6 mice at different ages [3 weeks (w), 8 w, 4 months (m), 9 m]. For each independent immunohistochemistry (IHC) analysis, we obtained the testes from 4 C57BL/6 mice at different ages (3 w, 8 w, 4 m, 9 m). Moreover, each experiment was repeated three times. All tissues were collected and weighed using the same method.

### IHC analysis

The testis and epididymis samples were extracted from C57BL/6 mice. The paraffin- embedded tissues were cut at 4 μm. Then, each section was dewaxed in xylene at 60°C and rehydrated in ethanol solutions. Endogenous peroxidase activity was blocked with 0.3% H_2_O_2_ solution for 10 min at room temperature. Next, the sections were incubated in citrate buffer solution at 100°C for 30 min and washed with PBS. The sections were blocked with 5% BSA for 20 min at 37°C and incubated with anti-B7-H3 antibody (diluted 1:200) overnight at 4°C. The sections were incubated with horseradish peroxidase-conjugated goat anti-rabbit IgG the next day. Then, the sections were counterstained with hematoxylin.

### Western blot

Each testis was cut into 1 mm^3^ pieces. Then, they were digested with type IV collagenase for 1 h at 37°C. After the tissues were homogenized in PBS, they were incubated with cell lysis buffer containing protease inhibitors on ice for 30 min. The samples were centrifuged at 14,000 X for 10 min at 4°C, and the supernatant was collected for western blot analysis. For SSCs, the cells were harvested with 0.25% trypsin and incubated with cell lysis buffer. The remaining steps were performed as described above. All samples were boiled with 6×SDS-PAGE sample loading buffer for 5 min at 95°C.

The proteins were separated using 10% SDS-PAGE and then transferred onto 0.45 mm polyvinylidene difluoride (PVDF) membranes. After the membranes were blocked in 5% fat-free milk/0.2% Tween for 1 h at room temperature, they were incubated with primary antibodies against B7-H3 (diluted 1:1000), P-PI3K (diluted 1:500), PI3K (diluted 1:1000), P-JNK1/2/3 (diluted 1:5000), P-ERK1/2 (diluted 1:1000), and GAPDH (diluted 1:1000) overnight at 4°C. The next day, the membranes were incubated with the goat anti-rabbit IgG secondary antibody for 1 h at room temperature. Then, detection was performed using the BeyoECL Plus substrate system.

### Isolation of mouse SSCs

SSCs were collected from the testes of C57BL/6 mice 7 days after birth by a 2-step enzymatic digestion, according to an existing protocol [35]. Briefly, we first removed the tunica albuginea from the testis to expose the seminiferous tubules. Then, the testes were incubated with 1 mg/ml type IV collagenase for 15 min at 37°C, followed by digestion with 0.25% trypsin for 10 min at 37°C. The testis cells were plated on a gelatin-coated plate overnight, and the floating cells were plated onto other plates. After two to three passages, the cells were transferred onto MEFs pretreated with mitomycin C.

### Culture of mouse SSCs and TM4

Mouse SSCs were maintained on mitomycin C-treated MEFs. The basal culture medium was high-glucose DMEM supplemented with B27, 25 μg/ml insulin, 100 μg/ml transferrin, 10^3^ U/ml recombinant murine LIF, 10 ng/ml recombinant murine GDNF, and 1% penicillin/streptomycin [32, 36]. Cells were cultured in an atmosphere of 5% CO_2_ at 37°C. The cells were passaged and split at a 1:2 ratio when they attained 70% to 80% confluence. The rest of the cells were discarded. TM4 cells were cultured in high-glucose DMEM in an atmosphere of 5% CO_2_ at 37°C.

### Flow cytometry assessment of C-kit and Oct-4 expression in mouse SSCs

Flow cytometry analysis of C-kit and Oct-4 expression was performed as described previously [37]. Briefly, SSCs were harvested with 0.25% trypsin and were then resuspended in PBS/FBS. The cells were incubated with an anti-C-kit antibody (diluted 1:100) and anti-Oct-4 antibody (diluted 1:50) for 20 min at 4°C.FITC-conjugated goat anti-rabbit IgG (diluted 1:100) and PE-conjugated goat anti-rat IgG (diluted 1:100) were used as secondary antibodies. The cells were then washed three times with PBS/FBS and analyzed using a FACScan Flow Cytometer (BD Biosciences, NJ). The controls were not treated with primary antibodies.

### Transwell co-culture of mouse SSCs and TM4

Mouse SSCs (1 × 10^4^) were seeded in high-glucose DMEM with supplements in the upper chamber of transwell inserts (1-μm size pore, Corning, Tewksbury, MA) in 24-well plates, and 5×10^4^ mitomycin C-pre-treated TM4 cells were seeded in the lower chamber. After co-culture for 6, 12, 24, 48, and 72 hours, the supernatants were collected for B7-H3 quantification, while the SSCs were harvested for proliferation or cell cycle assessments.

### Cell proliferation by CCK-8 assay

CCK-8 assays were used to determine the effect of B7-H3 on mouse SSC proliferation. Briefly, mouse SSCs plated at 8000 cells per well in triplicates in a 96-well plate were maintained in DMEM supplemented with different concentrations (0, 5, 10, 25 ng/ml) of recombinant mouse B7-H3 for various times (1, 6, 12, 24, 48, 72 h). At each time point, 10 μl CCK-8 solution was added to each well. Then, the cells were cultured in a humidified atmosphere of 5% CO_2_ at 37°C for 1 h. Finally, the absorbance of each sample was detected at 450 nm by a Thermo Multiskan Mk3 microplate reader (Labsystems, Helsinki, Finland).

### Cell cycle analysis by BrdU

For analysis of the role of B7-H3 in mouse SSC growth, SSCs were incubated with different concentrations of B7-H3 (0, 5, 10, 25 ng/mL) for 48 and 72 h. For analysis of the effects of P-PI3K on mouse SSC proliferation, SSCs were incubated with 10 ng/ml B7-H3 for 48 and 72 h after pretreating the cells with 10 μM of the inhibitor LY294002 for 6 h; the control groups were incubated with 0 and 10 ng/ml B7-H3 for 48 and 72 h. Cells were treated with 10 μM BrdU for 30 min before collection with 0.25% trypsin and were then fixed in 70% ethanol. DNA denaturation was performed by incubation with 2 M HCl/0.5% Triton X-100 for 30 min at 37°C. After the cells were neutralized with Na_2_B_4_O_7_ and washed with PBS, they were incubated with an anti-BrdU antibody (diluted 1:40) for 1 h. Then, the cells were incubated with Alexa Fluor 488-conjugated goat anti-rat IgG antibody (diluted 1:2000) for 30 min after centrifugation. Finally, the cells were resuspended with 20 μg/mL propidium iodide to stain the nucleic acids and analyzed by a FACScan Flow Cytometer (BD Biosciences, NJ) [38, 39].

### Statistical analysis

All analyses were performed using GraphPad Prism 6.0, and data are expressed as the mean±standard deviation. Statistical differences between groups were determined using one-way ANOVA followed by individual *t*-tests with Tukey's multiple comparison test. Groups with two factors (B7-H3 concentration and incubation time) were analyzed using a two-way ANOVA and Tukey's post test for multiple comparisons. Data were considered statistically significant at *P* < 0.05.

## SUPPLEMENTARY MATERIALS FIGURE



## Abbreviations

SSCs: Spermatogonial stem cells
PI3K: Phosphoinositide 3-kinase
IFN-γ: Interferon-γ
VEGF: Vascular endothelial growth factor
ERK: Extracellular signal-regulated kinases
JNK: C-Jun N-terminal kinase
SCF: Stem cell factor
p70S6K: p70 S6 kinase
GDNF: Glial cell line-derived neurotrophic factor
Ras: Rat sarcoma
MAPK: Mitogen-activated protein kinase
MEK: MAPK/ERK kinase
ROS: Reactive oxygen species
IHC: Immunohistochemistry
PVDF: Polyvinylidene difluoride
