# Supplementary material for: B7-H3 promoted proliferation of mouse spermatogonial stem cells via the PI3K signaling pathway
Source: Oncotarget. 2017 Dec 20;9(2):1542–52. doi: 10.18632/oncotarget.23457 (PMC5788580; doi:10.18632/oncotarget.23457)
Supplement: Supplementary file 1 [file oncotarget-09-1542-s001.pdf]

B7-H3 promoted proliferation of mouse spermatogonial stem cells *via* the PI3K signaling pathway

SUPPLEMENTARY MATERIALS

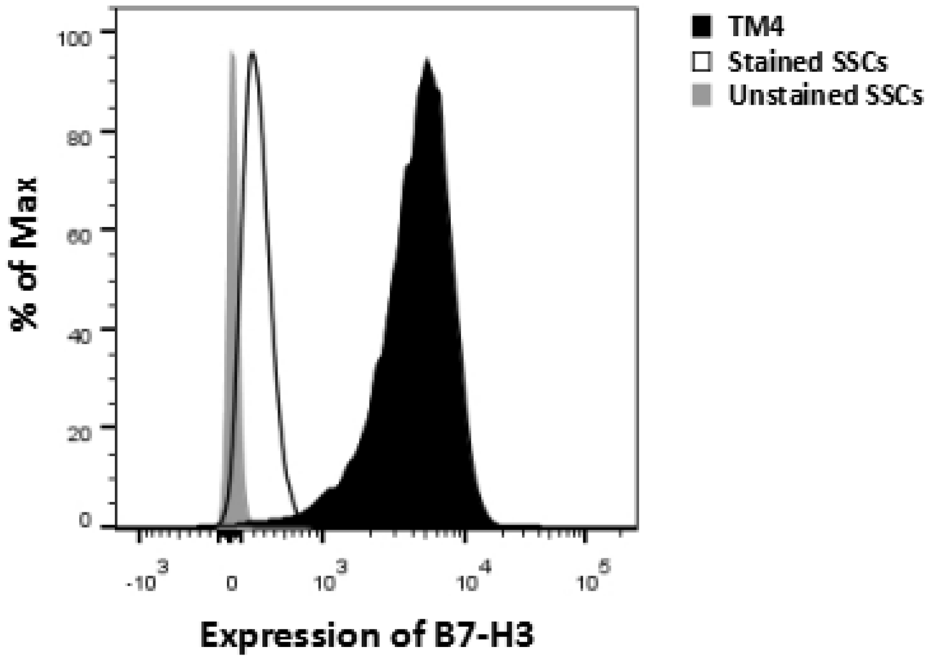

Supplemental Figure 1: The expression of B7-H3 on mouse SSCs. Mouse SSCs were unstained or stained with FITC-anti-B7-H3 antibody at 1:40 for 20 minutes at 4°C and then assessed for the expression of B7-H3 using flow cytometry. Mouse Sertoli TM4 cells were also assessed as a positive control.
